# A wildfire vulnerability index for buildings

**DOI:** 10.1038/s41598-022-10479-3

**Published:** 2022-04-16

**Authors:** M. Papathoma-Köhle, M. Schlögl, C. Garlichs, M. Diakakis, S. Mavroulis, S. Fuchs

**Affiliations:** 1 Department of Civil Engineering and Natural Hazards, University of Natural Resources and Life Sciences, 1190 Vienna, Austria; 2 Institute of Geography, University of Innsbruck, 6020 Innsbruck, Austria; 3 Faculty of Geology and Geoenvironment, School of Sciences, National and Kapodistrian University of Athens, Panepistimioupolis Zografou, 15784 Athens, Greece

**Keywords:** Environmental sciences, Natural hazards

## Abstract

Recent wildfire events (e.g. Mediterranean region, USA, and Australia) showed that this hazard poses a serious threat for wildland–urban interface (WUI) areas around the globe. Furthermore, recent events in regions where wildfire does not constitute a frequent hazard (e.g. Siberia, Scandinavia) indicated that the spatial pattern of wildfire risk might have significantly changed. To prepare for upcoming extreme events, it is critical for decision-makers to have a thorough understanding of the vulnerability of the built environment to wildfire. Building quality and design standards are important not only because building loss is costly but also because robust buildings may offer shelter when evacuation is not possible. However, studies aiming at the analysis of wildfire vulnerability for the built environment are limited. This paper presents an innovative solution for the vulnerability assessment to wildfires, making use of an all-relevant feature selection algorithm established on statistical relationships to develop a physical vulnerability index for buildings subject to wildfire. Data from a recent and systematically documented wildfire event in Greece (Mati, 2018) are used to select and weight the relevant indicators using a permutation-based automated feature selection based on random forests. Building characteristics including the structural type, the roof type, material and shape, the inclination of the ground, the surrounding vegetation, the material of the shutters and the ground covering were selected and formed into the index. The index may be used in other places in Europe and beyond, especially where no empirical data are available supporting decision-making and risk reduction of an emerging hazard amplified by climate change.

## Introduction

In the summer of 2021, the Mediterranean region experienced the worst heatwave over many decades leading to subsequent wildfires that destroyed excessive areas of forests, related industries, ecosystems but also buildings and infrastructure. In recent years, large forest fires have repeatedly affected various countries across Europe. However, 85% of the total burnt area in Europe is located in the EU Mediterranean region^1^. In the five most wildfire-prone EU Mediterranean countries (Portugal, Spain, France, Italy, and Greece) the average burnt area for the period 1996–2016 was more than 400,000 ha. More recently, only in Greece, more than 100,000 hectares were burnt in only two weeks in summer 2021 whereas the average burnt area during the same period (end of July–mid August) in the last 12 years (2008–2020) was 2750 hectares^2^. In 2021, the number of wildfires largely exceeded the number of fires in previous years in the region. In more detail, according to EFFIS (European Forest Fire Information System), the average number of forest fire events in Europe in the period 2008–2020 was 683, whereas only in 2021 at least 1766 events were recorded until August^3^.

Climate change does not necessarily affect the number of fires occurring in fire-prone countries or the extent of the burnt area^4–7^, but it does lead to warmer and drier conditions^8^. As a consequence, the length of fire weather periods is likely to increase^9^ and the occurrence of extreme events is likely to become more frequent^8,10^. In this context, extreme events result in considerable socioeconomic and environmental damages, such as the wildfires that occurred in Greece in 2007 and 2018. These so-called “mega-fires” cause the majority of damages although, they account only for 2% of the total number of fires^7^. Furthermore, the fire weather periods demonstrated an 18.7% increase in the period 1979–2013, and the affected area globally increased by more than 108%^9^. As a result, new regions have been and will be affected by increasing wildfire risk (e.g. Russia, Canada, Scandinavia)^9,10^. According to Komac et al.^10^, wildfire risk is expected to increase in Europe due to a combination of factors that are not limited to changes in climatic conditions. These factors include also human–environment interactions such as land-use changes, urbanisation, and focus on fire extinction rather than prevention strategies. In parts of Southern Europe 821 individuals have died between 1979 and 2016 from wildfires, a large portion of which in cases of entrapments in WUI areas^11^.

Risk quantification and visualisation of its spatial patterns are of paramount importance for the design of disaster risk reduction strategies that aim at the reduction of negative consequences caused by wildfires to the built environment, to the infrastructure, and most importantly to the safety of the population. As vulnerability is a key parameter of risk, decision-makers, authorities, and other stakeholders need tools that enable vulnerability analysis and consequently its reduction.

In natural hazards research, physical vulnerability is assessed through the use of matrices, indices, and functions^12^. The present study focuses on indices derived from indicators that are defined as “operational representations of a characteristic or quality of a system”^13,14^. The main advantage of indices and indicator-based methods is that complex issues are summarized facilitating in this way communication among stakeholders^15^ and towards the inhabitants of WUI areas. The development of an index, in general, incorporates a number of steps including indicator selection, weighting, and aggregation. The choice of the indicators depends on the nature of the assessment and the hazard under consideration. After the collection of the relevant data, the indicators have to be weighted and aggregated into an index. According to OECD^16^, the weighting and the aggregation of indicators to indices largely influences the resulting rankings^17^ and consequently decision making^18^. Despite the importance of weighting most of the indicator-based methods for vulnerability assessment use equal weighting and only a few are based on statistical weighting approaches^19^.

Indicator-based methods are common for the assessment of social vulnerability. Nevertheless, approaches based on indicators focusing on physical vulnerability have been developed for tsunamis^20^, floods^21^, and dynamic flooding^22^.

As far as wildfires are concerned, although the role of building characteristics to their overall vulnerability has been investigated^23–32^, very few studies^33–41^ (Table 1) have developed a systematic approach for the assessment of physical vulnerability (often as part of a risk assessment approach or combined with social vulnerability) that can be used as a basis for disaster risk reduction strategies. These studies focus on different scales, resolutions, and degrees of detail. However, the sets of indicators used concerning physical vulnerability are very limited (e.g. housing density, structural type). Moreover, very few advances can be seen in the weighting method of these indicators and their aggregation into an index. A short description of existing approaches, as well as their advantages and disadvantages, is provided in Table 1.

**Table 1:** Recent studies focusing on the physical vulnerability of buildings to wildfire.

Study	Geographical location	Short description of the method	Advantages and disadvantages
Caballero and Beltran (2003)^33^	Spain	Mapping of individual factors that affect vulnerability (e.g. surrounding vegetation)	*Advantage* information regarding the factors influencing vulnerability *Disadvantage* no reproducible holistic approach
Lampin-Maillet et al. (2010)^34^	France, Spain and Greece	A vulnerability index based on several factors: the factors have been grouped into four components (difficulty of extinction, demand for forest defence, demand of civil protection and territorial value)	*Advantage* many factors not directly connected with the interaction between the structure and the fire are concerned (e.g. population dependency on civil protection) *Disadvantage* the study is implemented at the regional scale and the unit of research is not the individual building but territories
Ganteaume and Jappiot (2014)^38^	France	Focus on risk; housing density, fence; and type of surrounding vegetation considered	*Advantage* very detailed analysis of surrounding vegetation types and flammability *Disadvantage* very few indicators on building characteristics
Penmann et al. (2015)^39^	Australia	As part of a Bayesian network the house condition is characterised based on surrounding vegetation, openings, gutters, ground covering, etc.)	*Advantage* very detailed information about the house is considered to identify its condition *Disadvantage* the method is not based on empirical data
Mhawey et al. (2017)^35^	Lebanon	Wildland urban interface building risk index (vulnerability assessment is part of it)	*Advantage* the results can be used for cost–benefit analysis *Disadvantage* only the value of the buildings is considered
Papakosta et al. (2017)^40^	Cyprus	Bayesian networks for wildfire risk. Factors considered for the vulnerability of houses: housing stock (single houses, rowhouses, apartments), construction type (material/roof), housing density and construction value	*Advantage* focus on economic costs, rather detailed information on the wildfire process *Disadvantage* no detailed information on buildings
Oliveira et al. (2018)^36^	Portugal, Italy (Sardinia), France (Corsica)	Weighted index	*Advantage* a multi-dimensional approach *Disadvantage* only building and road density is considered for the physical vulnerability
Ghorbanzadeh et al. (2018)^37^	Iran	The approach is applied on a regional scale and considers only spatial characteristics of the built-up area (e.g. distance to roads) and not characteristics of individual buildings	*Advantage* social and infrastructure vulnerability *Disadvantage* regional scale. The approach cannot be used for the vulnerability reduction of individual buildings
Andersen and Sugg (2019)^41^	California (USA)	Social and physical vulnerability assessment. Consideration of factors for the assessment for physical vulnerability: fuel (biomass, forest), topography (elevation, slope), climate and development (population and road density)	*Advantage* combination of physical and social vulnerability to wildfires *Disadvantage* no vulnerability indicators regarding building characteristics

We consider the use of indices for the assessment of physical vulnerability useful because they are directly related to the building characteristics that may change reducing in this way the vulnerability of a structure in a cost-effective way. Indices do not make direct use of empirical data (once they have been developed) and can be applied in areas without any historical record of events. Moreover, during the development of an index, we have a closer look at the interaction between buildings and the hazardous process (in this case wildfire) which is beneficial knowledge for building back better and designing building codes in the WUI. Existing methods using indicators (listed in Table 1) however, are often not in local scale using buildings as the research unit and do not use detailed indicators.

We present herein a method for the development of a Physical Vulnerability Index (PVI) for buildings subject to wildfire that considers many different building characteristics and their surroundings and uses weighting based on statistical methods (all-relevant Boruta feature selection). One of the main advantages of the method is its predictive capacity and the ability, once established, to indicate houses with high loss potential in areas susceptible to wildfires in the future.

In more detail, the present study incorporates the following innovative aspects:

- *Focus on an emerging hazard* Given the expected increase in wildfire events in the future, the present study deals with an emerging hazard. Wildfires are expected to increase in the future also in areas where they were not common in the past. Decision makers in these areas are in need of tools and strategies for wildfire risk reduction.
- *Focus on vulnerability* The study focuses on the impact of wildfire on elements at risk rather than its properties as a natural process (fire ignition, propagation, forecasting, modelling, etc.) exploring at the same time possibilities for adaptive solutions.
- *Focus on indices* A Physical Vulnerability Index (PVI) is developed for wildfires, giving deeper insights into the interaction between the building envelope and hazard dynamics demonstrating the value of indicator-based methods for the assessment of physical vulnerability.
- *Focus on selection and weighting of indicators* Boruta, an all-relevant feature selection algorithm, is applied to obtain the relevant indicators that may be considered in the construction of a new index, reducing in this way the required amount of data. Boruta feature selection is also used to support the weighting of the indicators based on the importance of each indicator in explaining the degree of damage.
- *Focus on application and transferability* The presented Physical Vulnerability Index (PVI) for wildfire is presented which can be used in other places in Europe and beyond that may experience similar challenges in the near future due to climate change. The application of the PVI is carried out in a very typical Mediterranean environment both in terms of (a) vegetation type (i.e. pine trees) and (b) dominant building types. Taking into account the abundance of these characteristics in the Mediterranean region and beyond, it is reasonable to assume that the PVI can be applied in study areas with similar housing type and environmental context. In other areas in the world, the index has to be modified to consider existing structural types and building particularities but the methodological steps leading to its development can be transferred without modifications.
- Furthermore, the PVI can be used to support prevention and preparedness at different levels (homeowners, local authorities) and by different stakeholders (insurance companies, emergency services, etc.).

**Figure 1:**
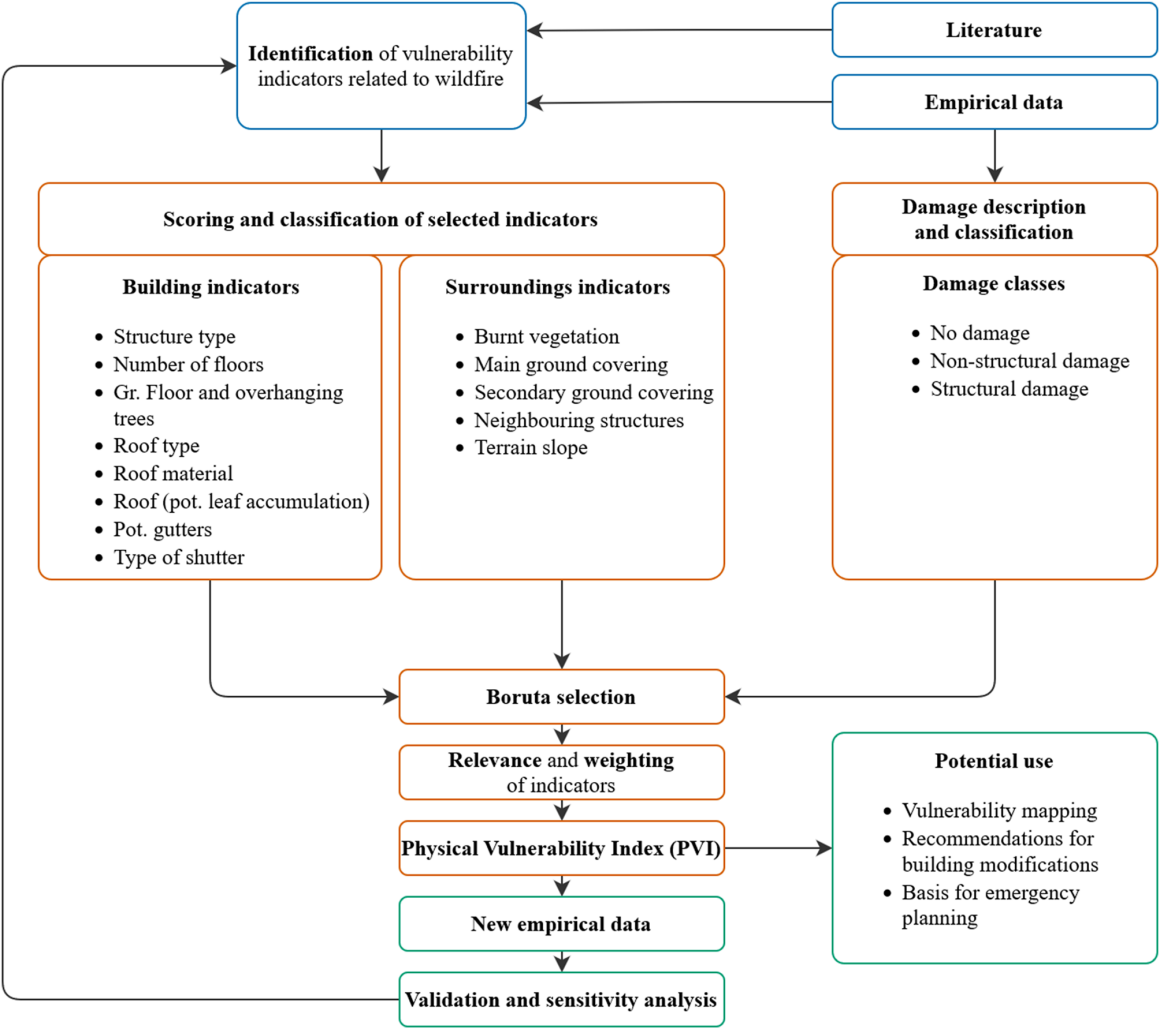
The methodological flow of the study (showing also—in green—possible future steps that are not included in the present paper).

The aim of this paper is to describe the adaptation of an approach that has originally been established for assessing physical vulnerability for tsunamis and dynamic flooding to another hazard type (wildfire). Specifically, based on empirical data from a recent event, the procedure followed for the making of the new PVI for wildfires is similar to the one that has been followed for tsunamis^20^ and dynamic flooding^22^ (Fig. 1). The present study focuses on the process of indicator selection and weighting based on the importance of building characteristics being tested using empirical high-resolution data from a recent catastrophic event in Greece (Mati, 2018), a typical WUI area of the north Mediterranean coastal region. The study attempts the development of a Physical Vulnerability Index and makes a significant step towards the understanding of the interaction between buildings and wildfire. For this reason, the advantages and limitations of the development of the PVI as well as possible future research directions are also discussed in the following sections.

## Results

### The case study area and the event of 2018

**Figure 2:**
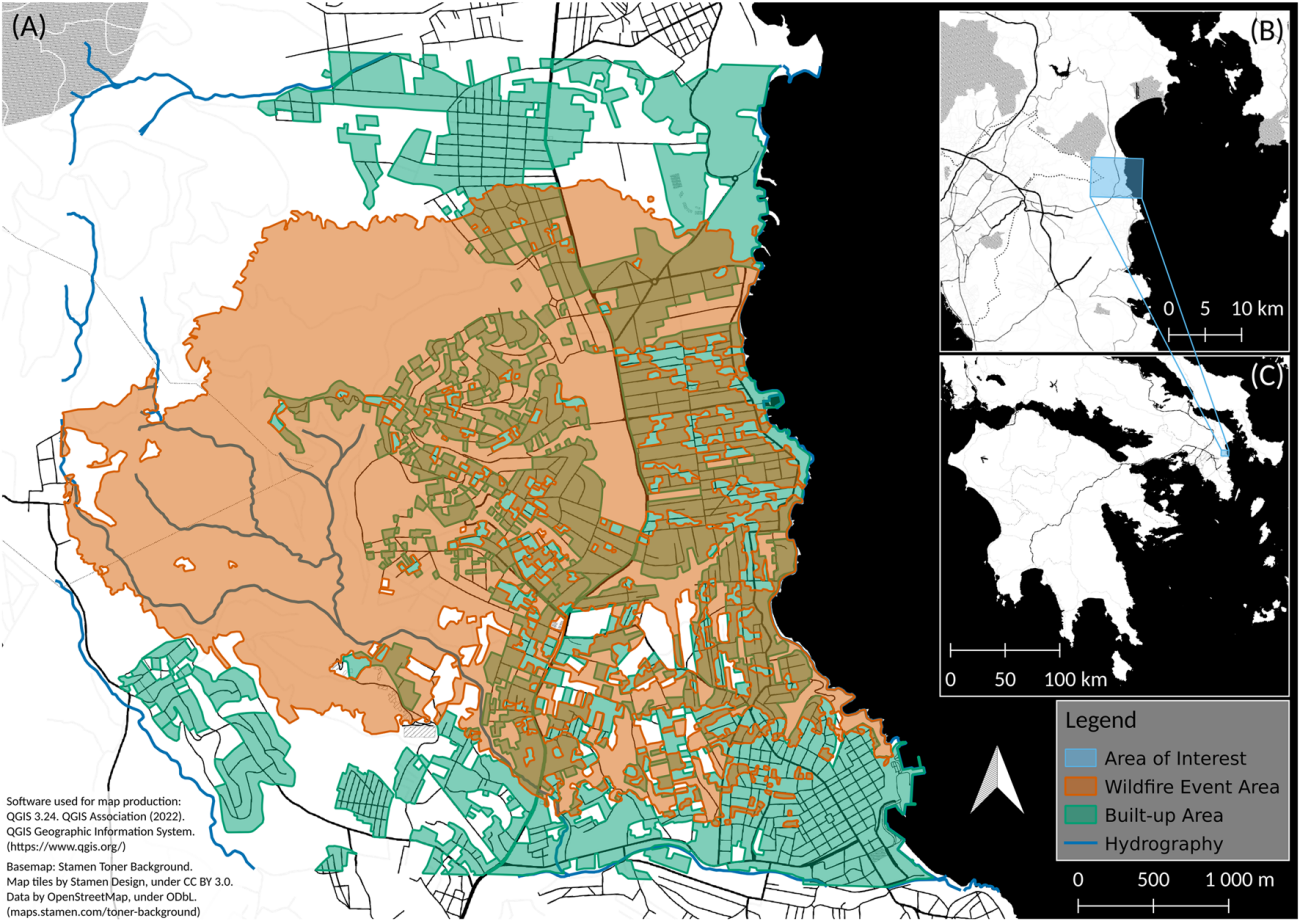
Map of the area under study showing the extent of the wildfire as of 26 July 2018 16:40 (UTC). The main map (**A**) shows the area affected by the wildfire event and the built-up area. Data is taken from the Copernicus Emergency Management Service (EMSR300)^54^. The inlets show the area of interest in a broader context. (**B**) Shows the location of the study area around Mati on the east coast of Attica. (**C**) Displays the location of the area of interest in the Western Aegean.

The empirical data used for the analysis for the vulnerability indicators herein derive from a recent wildfire event which occurred in July 2018 in Mati (Attica region, Greece) very close to the Greek capital Athens (Fig. 2). Mati, a typical WUI area, was a popular summer holiday destination for Athenians that had their holiday homes located in the pine forest which occupied the majority of the coastal segment. On July 23, 2018 a crown fire was initiated on the mountain Penteli (5.2 km west from the coast) driven by very high winds (90–120 km/h) and rapidly reached the resort of Mati^42–45^ in less than two hours from ignition^10^. The synergy of factors including the characteristics of the fire (speed), lack of coordination of operations, underestimation of the seriousness of the situation at the beginning, and the chaotic intermix of housing and dense vegetation (typical WUI) led to an inadequate response. Furthermore, the challenging topography in combination with the settlement configuration (informal housing, poor planning) resulted in difficulties in evacuation and eventually in many casualties and loss of buildings and infrastructure. The fire resulted in 102 deaths but also the burning of 1400 ha of land, 1200 buildings, and 300 vehicles.

### The making of the PVI

A list of indicators (Table 2) was derived based on expert judgment, an extensive literature review and a review of damage documentation of past events, and past vulnerability assessment studies focusing on the impact of wildfires on buildings. It has to be noted that until now previous literature identifies certain building characteristics using documentary evidence, expert judgment, and empirical data but not through exploring statistical significance^26^.

**Table 2:** The scoring of the indicators collected in the field.

Indicator	Abbreviation	1	2	3	4	5
**Building**						
Structure type	STR_T	Stone construction with load-bearing masonry	RC structure	Light metal frame and plasterboard in gaps and mixed materials	Wooden structure	Mobile home (container)
No of floors	N_FLO	1				2; 3; 4; 5
Ground floor and overhanging tree	B_HTRE	No. of floor more than ground floor			Ground floor building with tangent trees	Ground floor building with overhanging tree
Roof type	ROF_T	/	–	Tireless flat and shed	Dual-, three- or four-pitched roof (gable, hipped)	layered (combines different designs according to the floor plan of the building)
Roof material	ROF_M	Concrete slab with tiles	Concrete slab	Metal roof with ceramic or metallic tiles (elenit)	Wooden roof with ceramic tiles	Wooden roof with metal tiles
Roof (potential leaf accumulation), see Table 3	ROF_LA	1	2	3	4	5
Potential gutters (leaf accumulation), see Table 4	P_GUT	1	2	3	4	5
Type of shutter	SHUT		–	Aluminium	Mixed	Wood and plastic
**Surroundings**						
Burnt vegetation	VEG	No vegetation	Vegetation within 20 m	Bushes within 20 m	Tangent bushes and trees within 20 m	Hanging trees and tangent trees
Main ground covering	M_GC	Bricks	Slate slab and pebbles and chippings	Concrete	–	Natural soil without coating
Secondary ground covering	S_GC	Bricks	Slate slab and pebbles and chippings	Concrete	–	Natural soil without coating
Neighbouring structures	NEIGH	Empty surrounding space	–	–	–	Direct contact to neighbouring buildings
Terrain slope	SLOP	Mild (0–5%)	–	Average (5–25%)	–	Intense (> 25%)

### Selection and weighting of vulnerability indicators

A literature review of studies focusing on characteristics of buildings related to their vulnerability to wildfire in various wildfire-prone countries was used as a basis for the development of a list of indicators (Table 2). The characteristics of buildings and their surroundings as well their location have been reported from several studies focusing on specific areas around the world such as the USA^28,29,31,46–48^, Australia^39,49–51^, the Mediterranean^23^, or other regions^27^. Some of the studies focus directly on vulnerability analysis^47^, others offer retrofitting options^52^ and building standards^24^ or evaluate the effects of mitigation measures or urban planning^53^. All studies give a very good picture of the interaction of the fire with the building envelope focusing on the surrounding vegetation, the location of the building, the surrounding buildings that may cause shielding effects as well as characteristics of the structure related mainly to materials, openings and the roof. A selection of indicators associated with the vulnerability of the buildings to wildfire, that can be found in the literature is demonstrated in Table 7 (“Methods” section). Not all of these indicators were collected for the study area since some of them were not relevant due to the dominant architectural style in the area or others were not possible to be collected due to the timing (data collection occurred after the event and many features were already destroyed).

The area under study was the affected (burnt) area in the settlement of Mati (Fig. 2) which occupied approx. 7.8 km^2^. Despite the fact that more damaged buildings were located on the mountain, the majority of human losses was reported in the densely populated coastal settlement of Mati. The indicators were collected during post-fire field survey (a few hours after the fire had gone out).

In more detail, all the indicators demonstrated in Table 2 and damage patterns in Table 5 were collected for a total of 423 buildings in the study area. For each of these buildings, the degree of damage (a description of the condition of the building following the disastrous event) was documented, and due to the lack of information concerning the actual monetary damage, each building was assigned to one of the three damage categories (Table 5). The scores for each indicator are shown in Table 2. Tables 3 and 4 are explanatory to Table 2.

**Table 3:** Potential leaf accumulation on the roof.

	Roughness of roof material		
		Concrete	Metal and ceramic tiles
Complexity of roof shape			
	Tireless flat and shed	1	2
	Dual-, three- and four-pitched roof (gable, hipped)	2	4
	Layered (combines different designs according to the floor plan of the building)	3	5

**Table 4:** Potential leaf accumulation in gutters.

Roof shape	Gutters	Potential leaf accumulation in gutters
Tireless flat	0 (surface)	1
Shed	1	1
Dual-pitched roof (gable)	2	2
Three-pitched roof	3	3
Four-pitched roof (hipped)	4	4
Layered (combines different designs according to the floor plan of the building)	> 4	5

**Table 5:** The classification of fire induced damage into three categories according to the damage description (Photos by Michalis Diakakis and Spyridon Mavroulis, from Lekkas et al.^56^).

Damage degree and related description	Photo documentation
*No damage* The building is located within the burnt area. However, no damage (structural or non-structural) has been observed and recorded. (damage degree 1)	
*No structural damage* The building is located within the burnt area and non-structural damage (e.g. burnt building features such as tents, damage of the outside walls, melting of aluminium shutters and frames etc.) has been recorded. (damage degree 2)	
*Structural damage* The structural elements of the building including the load-bearing frame suffered heavy or severe damage comprising partial or total collapse of the structure. Thus, the building cannot be rehabilitated and has to be demolished. (damage degree 3)	

**Figure 3:**
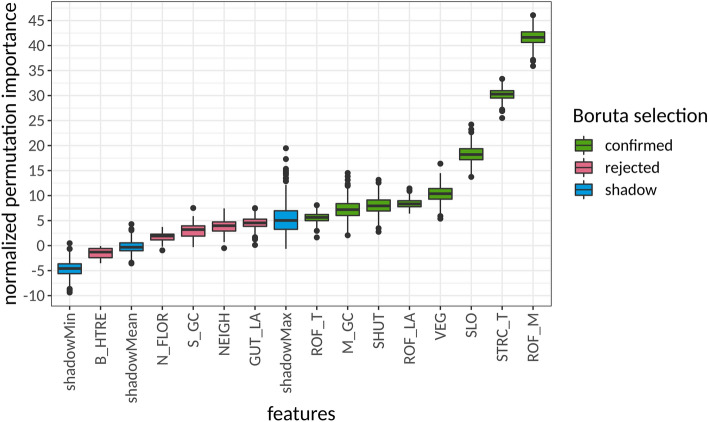
Results of the Boruta selection for indicators related to the vulnerability of buildings to wildfire.

The feature importance of each indicator on the degree of loss for each building was obtained using Boruta selection. In more detail, the random-forest-based all-relevant feature selection algorithm Boruta^55^ was used to estimate the feature importance of each indicator on the degree of damage*.* The Boruta selection pointed out: (a) the indicators that can explain the damage and may be used in the making of a PVI for wildfire and (b) the relative importance of these indicators in the physical vulnerability of the buildings to wildfire which may be used for the weighting of these indicators before their aggregation to an index (Fig. 3). The feature selection revealed five indicators that were rejected due to lack of explanatory power, whereas eight indicators performed better than their randomly permuted duplicates (shadow features) in explaining the degree of damage (Table 6).

**Table 6:** Relevant indicators to be considered in the index and their weights.

Code		Indicator	Weight	
*I*_*1*_	ROF_M	Roof material	*w*_1_	33%
*I*_*2*_	STRC_T	Structural type	*w*_2_	23%
*I*_*3*_	SLOP	Slope (Terrain)	*w*_3_	14%
*I*_*4*_	VEG	Vegetation	*w*_4_	8%
*I*_*5*_	ROF_LA	Roof-leaf accumulation	*w*_5_	6%
*I*_*6*_	SHUT	Shutter material	*w*_6_	6%
*I*_*7*_	M_GC	Main ground covering	*w*_7_	5%
*I*_*8*_	ROT_T	Roof type	*w*_8_	4%

The indicators and their associated relevance according to the Boruta selection can be seen in Fig. 3 and are listed in Table 6. The indicators were weighted (Table 6) using the relative contribution of each relevant feature to the total importance of all relevant features. This was derived by the division of the respective features’ median importance by the sum of median importance for all relevant features. Based on these results, the PVI Eq. (1) has been calculated for each building in the area.

## Discussion and conclusions

This paper describes the adaptation of a Physical Vulnerability Index (PVI) for buildings subject to the impact of wildfires. The index aims at covering the gap of previous studies that have not explored until now the statistical significance of vulnerability indicators of buildings subject to wildfire^26^.The development of the index is based on the statistical analysis of the relationship between different building characteristics and their immediate surroundings (vulnerability indicators) as well as the fire-induced damage degree. The Boruta feature selection was used to identify all relevant indicators that should be included in the index.

Based on evidence presented in previous studies associating certain building attributes to their susceptibility to fire (even with poor statistical confirmation), we collected various building data from the study area. Through the examination with Boruta algorithm some of these attributes were shown to have statistical association with the level of fire-induced damage indicating their role in the building’s vulnerability. Roof material showed the strongest association, partially confirming previous studies^23^ suggesting that the roof is one of the weakest points of a building.

Along with the roof material, the structural type and the terrain slope were the most relevant of all the indicators. Nevertheless, according to the results of the Boruta selection the main ground cover, the potential for leaf accumulation on the roof, the shutters, and the surrounding vegetation do also play a statistically significant role, confirming certain literature hypotheses^23,26,27,29,57–59^. Among the indicators that were not found to be significant were the secondary ground cover, the number of floors, the leaf accumulation in gutters, and the combination of the number of floors and neighbouring vegetation. Finally, the presence of neighbouring buildings, which has been very often reported in the literature as a very important factor for the physical vulnerability of buildings to wildfire^28,34^, has not been found to be relevant. The reason may be that most buildings had neighbouring structures (given the dense building settlement in Mati) and, for this reason, all the buildings had the same scoring as far as this particular indicator is concerned. Consequently, the specific indicator may not contain enough discriminatory information. Nevertheless, the index in its present form can still indicate the relative vulnerability of the buildings.

The PVI may demonstrate the presence of clusters of vulnerable buildings that may be used to guide local authorities and spatial planners to certain actions that may reduce the risk of future wildfire events. The chosen classification for the vulnerability categories method has the potential to influence the decision-making accordingly*.*

**Figure 4:**
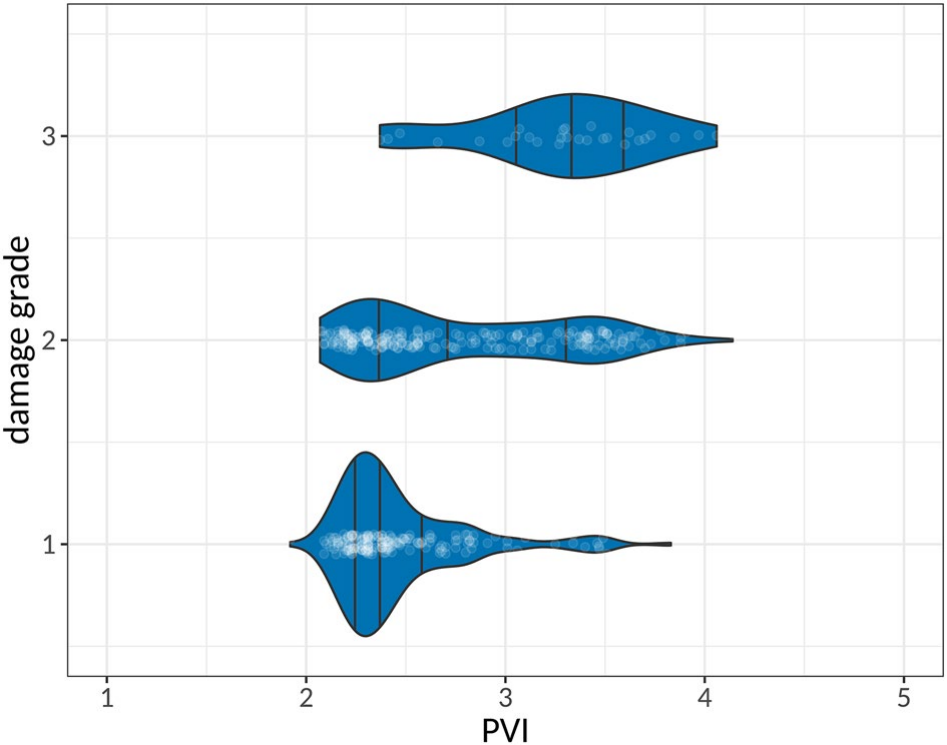
The relationship between the damage grade of a building and the assigned PVI. Vertical lines within the violin plots indicate the three empirical quartiles of the PVI for each damage class. The number of buildings is 167, 212, and 28 for damage grades 1, 2, and 3, respectively.

The PVI was calculated for each building in the case study area. To validate the PVI, the relationship between the resulting index and the damage grade per building is shown in Fig. 4.

Figure 4 shows that the higher the damage grade of the building is, the higher is its PVI. Non-parametric rank correlation tests based on both Spearman’s *ρ* (p = 3.465e−15) and Kendall’s *τ* (p = 3.25e−14) yield highly significant results, allowing to reject the null hypothesis of the true rank correlation coefficients being equal to 0. Consequently, results confirm a statistically significant positive ordinal association between the PVI and the observed damage grade. The strength of the relationship can be quantified based on analysing concordant and discortant pairs of PVI values and damage grades. Resulting estimates (and the corresponding 95% confidence interval) for rank correlation metrics are γ = 0.4 (0.32, 0.49) τ_C_ = 0.3 (0.23, 0.36) for Goodman and Kruskal's γ and Stuart–Kendall τ_C_, respectively.

**Figure 5:**
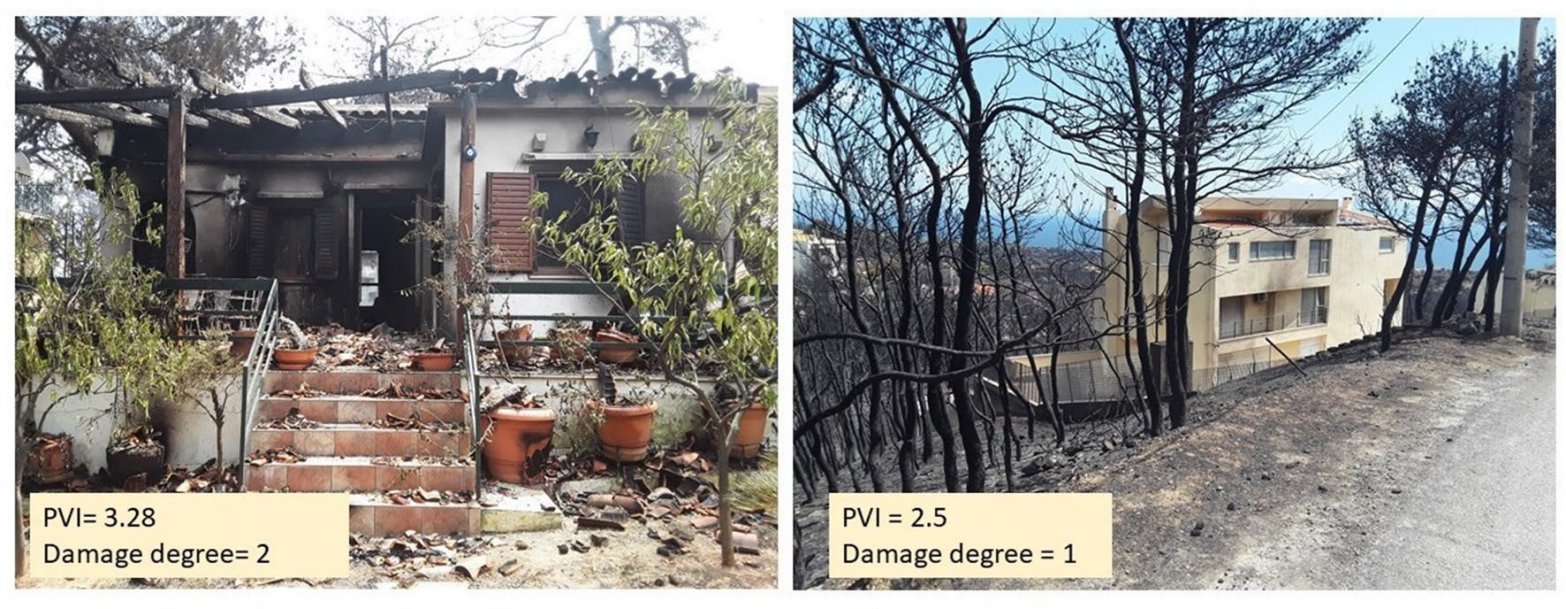
Burnt buildings in Mati (Photos by Michalis Diakakis and Spyridon Mavroulis).

In other words, buildings with high physical vulnerability experienced damages of a higher grade. This can also be seen in Fig. 5. The individual cases that do not follow this rule may be explained by factors that were not examined in this study (e.g. building interior elements) and/or other non-intrinsic building parameters such as fire temperature, wind characteristics, fuel loads, and others. Differences in the process itself (direction of the wildfire) on individual buildings may also explain these differences.

In this paper, the stepwise development of an index to assess the physical vulnerability of buildings to wildfires in the Mediterranean context has been described. The approach and the index itself demonstrates advantages in comparison to existing studies (Table 1) because it includes a very detailed set of indicators at a very local scale. The index is based on (very recent) empirical data. The indicator selection and weighting method is very sophisticated targeting multiple users delivering important information that can be used in many different ways. The results may be beneficial and may guide recommendations for building codes for new buildings in the WUI targeting specific building features such as the roof material and shape, the material of shutters and the structural type. Local authorities may also issue recommendations to homeowners regarding the choice and maintenance of surrounding vegetation and the cleaning of the area surrounding their property. However, it is important to consider that the study presented herein was subject to some assumptions and limitations: (a) the data were collected mainly in the field after the event; (b) the damage degree was recorded in the field (post-fire) and expressed in distinctive damage groups defined by qualitative (damaged elements description and building condition) and not monetary terms; (c) factors associated with fire and fuel load parameters have not been taken into consideration in the current analysis, (d) transferring the index to another case study with different houses and environmental context (vegetation, terrain, etc.) would require modifications regarding the set of indicators and their weighting. Nevertheless, the methodological approach would be exactly the same (if the required empirical data are available) showing clearly that the methodology itself is transferable.

Moreover, the PVI does not consider particular wildfire attributes such as flame height, temperature etc. These attributes are highly dynamic, very localized and difficult to measure and observe during or after the fire event. In the present study, the index was developed solely on the characteristics of buildings and immediate surroundings, reflecting in essence the intrinsic susceptibility of buildings to fire damages, an approach adapted from previous studies^22^.

The application of the PVI is developed in a very typical Mediterranean environment both in terms of (a) vegetation type (i.e. pine trees) and (b) building types (including reinforced concrete buildings with infill walls, buildings with masonry load-bearing walls and mixed types, with a variety of roof and shutter types, etc.) encompassing a large variety of characteristics. Taking into account the abundance of these characteristics in the Mediterranean region and beyond, it is assumed that the PVI can be applied in other study areas. However, its application should be considered with caution in areas with entirely different building construction and geoenvironmental context (e.g. entirely wooden structures), where it would be necessary to reexamine the statistical relationships and the weighting of different parameters. Expansion of future research such areas would benefit our understanding on susceptibility of buildings to fire impacts and enrich the transferability of the PVI.

In summary, the development of the index leads to the following outcomes:

- The results of the Boruta selection confirmed certain literature hypotheses considering building characteristics that affect a building’s vulnerability to wildfire. Certain building features, however, seem to play a less significant role in the vulnerability of a building to wildfire than initially expected.
- The application of the index in Mati (Greece) showed that in the majority of the cases buildings with high PVI experienced significant damage during the 2018 wildfire, showing a high degree of association.
- Areas with similar types of buildings can make use of the presented index and weighting even if they have not experienced wildfire events in the recent past*.* The transfer of the method in areas with substantially different architecture still needs to be tested and further investigated.

The PVI for buildings subject to wildfire may be used by decision-makers giving an overview of the vulnerability of buildings at the local level, supporting in this way evacuation planning. Furthermore, it can be the basis for local adaptation measures and reinforcement of buildings that can support shelter-in-place^25,60,61^.

The present study opens the way for future research including the following:

- The index considers building characteristics but not the intensity or other characteristics of the fire or other physical processes that may amplify the impact on the buildings. More research is needed on normalizing fire impacts by accounting for the variability of fire intensity as far as its impact on the built environment is concerned and including it in the index. However, variables expressed in the current study such as the presence of vegetation near a structure encompass partly these factors.
- The results of the method have to be validated following a similar event in the future in a similar environmental but different architectural context. Future events may be used as a source of additional data that will update the weighting of the indicators and will demonstrate the transferability of the index.
- More detailed information regarding the damage (e.g. monetary loss instead of damage degrees) may lead to more reliable results of the Boruta selection.
- An interesting evolvement of PVI could be the transferability of the method in areas with different architecture and housing design (e.g. the European Alps).
- The making of an index requires detailed data that are not always available. More research can be done focusing on innovative ways for data collection, including approaches of citizen science.
- Alternative uses of the PVI should be investigated and tested. For example, following the reconstruction of a burnt settlement the application of the index can demonstrate the BBB (Build Back Better) by comparing the index results with the PVI before the event.

## Methods

In the following paragraphs the development of the PVI is described following the recommended steps outlined in the Handbook on Constructing Composite Indicators^16^.

### Theoretical framework

The development of the PVI should be guided by a theoretical framework that also includes the understanding of the vulnerability concept. The acceptable definition of vulnerability for this study is the one proposed by UNISDR^62^. Hence, vulnerability is “the conditions determined by physical, social, economic and environmental factors or processes which increase the susceptibility of an individual, a community, assets or systems to the impacts of hazards”. It is clear that in the present study the focus is on the physical conditions that increase the susceptibility of buildings to the impact of wildfires and on this basis the indicators will be selected.

### Selection of variables

The presented approach is based on previous methods used for the assessment of the physical vulnerability of buildings subject to tsunamis^20^ and dynamic flooding^22^. A literature review showed the factors and characteristics that have been highlighted or empirically associated with the vulnerability of buildings (Table 7). It was, however, impossible to collect all these indicators for each building on the case study partly because the information was not available and partly because the data collection took place after the event when some features of the buildings were already destroyed (characteristics of the interior such as curtains, upholstery, rugs, etc.). Furthermore, some of the characteristics listed in Table 7 are not common in the Greek architecture (e.g. attic) and others were not available since the buildings were not georeferenced (distance to the forest, distance to other buildings). All the co-authors of the present paper (six experts) were involved in the final indicator selection. The 13 indicators considered in this study are shown in Table 2.

**Table 7:** Factors related to the physical vulnerability of buildings to wildfire from the literature.

	Building part	Indicators	Reference
1	Envelope	Building use	Ghorbanzadeh et al. (2019)^37^
2		Building substance*	Laranjeira and Cruz (2014)^27^, Syphard et al. (2017)^31^ and Papalou and Baros (2019)^45^
3		Building façade/cladding	Laranjeira and Cruz (2014)^27^
4		Exterior subfloor system	Laranjeira and Cruz (2014)^27^
5		Roof material*	Laranjeira and Cruz (2014)^27^ and Vacca et al. (2020)^57^
6		Roof type (complexity)*	Laranjeira and Cruz (2014)^27^ and Vacca et al. (2020)^57^
7		Shutter*	Laranjeira and Cruz (2014)^27^
8		Door	Laranjeira and Cruz (2014)^27^
9		Window	Laranjeira and Cruz (2014)^27^, Papalou and Baros (2019)^45^
10		Type of window glass	Blanchi and Leonard (2005)^58^ and Vacca et al. (2020)^57^
11		Window size	Ramsay et al. (1996)^63^
12		Window frame	Laranjeira and Cruz (2014)^27^
13		Number windows/doors	Ramsay et al. (1996)^63^
14		Vents	Xanthopoulos (2011)^64^, Laranjeira and Cruz (2014)^27^, Syphard and Keeley (2019)^32^ and Vacca et al. (2020)^57^,
15		Covered vents (mesh)	FEMA (2008)^65^
16		Metal flywire screen	Laranjeira and Cruz (2014)^27^
17		Attic floor	Laranjeira and Cruz (2014)^27^
18		Chimney and stovepipes	Xanthopoulos (2011)^64^ and FEMA (2008)^65^
19		Stored materials (wood in the garden or under the patio)	Maranghides et al. (2013)^26^
20		Heat release rate of materials	Vacca et al. (2020)^57^
21		Water and gas supply pipes	Australian Standards (2009)^24^
22		Verandas and decks (material)	Australian Standards (2009)^24^
23		Housing density (isolated, scattered, dense clusters)	Lampin-Maillet (2010)^34^ and Alexandre et al. (2016)^28^
24		Evacuation possibilities (e.g. two exits)	Maranghides et al. (2013)^26^
25	Interior	Curtain material (e.g. nylon)	Laranjeira and Cruz (2014)^27^
26		Rugs	Laranjeira and Cruz (2014)^27^
27		Upholstery	Laranjeira and Cruz (2014)^27^
28		Polyurethane furnishing	Laranjeira and Cruz (2014)^27^
29		Timber furnishing	Laranjeira and Cruz (2014)^27^
30	Surroundings	Timber deck or porch	Laranjeira and Cruz (2014)^27^
31		Skirting or railing	Laranjeira and Cruz (2014)^27^
32		Garden furniture	Laranjeira and Cruz (2014)^27^
33		Fence, gravel border	Laranjeira and Cruz (2014)^27^
34		Garage, shed	Laranjeira and Cruz (2014)^27^ and Vacca et al. (2020)^57^
35		Balcony	FEMA (2008)^65^
36		Slope of land (> 10°)*	Quarles et al. (2013)^66^, Maranghides et al. (2013)^26^, Alexandre et al. (2016)^28^ and Institute for Business and Home Safety (IBHS) (2017)^67^
37		Terrain location	Maranghides et al. (2013)^26^ and Vacca et al. (2020)^57^
38		Neighbouring building*	Laranjeira and Cruz (2014)^27^ and Institute for Business and Home Safety (IBHS) (2017)^67^
39		Distance to building (settlement density)	Laranjeira and Cruz (2014)^27^
40		Distance to vegetation*	Ramsay et al. (1996)^63^, Cohen (2000)^68^, Leonard and Bowditch (2003)^69^, Mitchell and Patashinik (2007)^70^ and Laranjeira and Cruz (2014)^27^
41		Dead vegetation	Laranjeira and Cruz (2014)^27^
42		Vegetation type	Ramsay et al. (1996)^63^, Blanchi and Leonard (2005)^58^, FEMA (2008)^71^, Leonard et al. (2009)^59^, Laranjeira and Cruz (2014)^27^ and Vacca et al. (2020)^57^
43		Vegetation condition	Foote et al. (1991)^72^, FEMA (2008)^65^
44		Vegetation level of maintenance	Vacca et al. (2020)^57^
45		Overhanging tree*	Laranjeira and Cruz (2014)^27^
46		Vegetation density	Laranjeira and Cruz (2014)^27^
47		Distance edge of forest	Laranjeira and Cruz (2014)^27^
48		Ground covered vegetation*	Ramsay et al. (1996)^63^, Blanchi et al. (2006)^58^, FEMA (2008)^71^, Leonard et al. (2009)^59^, and Laranjeira and Cruz (2014)^42^
49		Distance to power lines	Xanthopoulos (2004, 2011)^23,64^
50	Emergency response and others	Distance to fire stations	Xanthopoulos (2004, 2011)^23,64^
51		Water source	Xanthopoulos (2004, 2011)^23,64^
52		Address	Xanthopoulos (2004, 2011)^23,64^
53		Escape routes	Xanthopoulos (2004, 2011)^23,64^
54		Road network condition	Xanthopoulos (2004, 2011)^23,64^
55		Accessibility	Xanthopoulos (2004, 2011)^23,64^
56		Spatial planning of the settlement	Galiana-Martin (2017)^30^

*Indicators used also in the present study.

### Scoring and normalization of data

The indicators were collected as categorical, numeric or binary raw data. Nevertheless, each indicator was given a score from 1 to 5 (Table 2) which indicates the degree to which it may contribute to the physical vulnerability of a building and allows the comparison with the other indicators (normalization).

### Weighting and aggregation

Although weighting is a very important step in the development of indices, equal weighting is the most common approach used in the literature. However, assuming equal weights across all indicators is a strong proposition (and most likely a severe simplification), which does not reflect the underlying complex relationships. Different weighting methods may lead to different outcomes that consequently will influence decision making^18^. Consequently, the choice of an objective and reproducible weighting method is of core importance with respect to reliable results. While expert judgment methods are used very often, statistical methods are employed comparably seldom to determine the weights of indicators. In the present study, we employ a permutation-based statistical testing strategy called Boruta^55^. At its core, Boruta is based on an assessment of the standardized and normalized accuracy loss (Z score) in random forest models. By performing two-sided tests of equality between the actual indicators and randomly permuted copies of these indicators (“shadow attributes”) using a 99% confidence level, the indicators get flagged as either important or unimportant in an iterative fashion. Important indicators are relevant in a sense that these features do show significantly more information content than the randomly permuted (and thus nonsensical) shadow attributes. The results of Boruta feature selection are shown in Fig. 3.

To calculate the PVI for wildfire the following equation was used:

1 $$PVI={\sum\nolimits}_{b=1}^{B}\left({w}_{b}{I}_{b}\right),$$

where PVI indicates the Physical Vulnerability Index, *I* represents the indicators used, *B* is the total number of indicators, and *w* their weight. The weights (${\mathrm{w}}_{\mathrm{b}}$) are derived dividing the respective features’ median importance ($\stackrel{\sim }{{\mathrm{f}}_{\mathrm{b}}}$) by the sum of median importances for all relevant features:

2 $${\mathrm{w}}_{\mathrm{b}}=\frac{\stackrel{\sim }{{\mathrm{f}}_{\mathrm{b}}}}{{\sum }_{\mathrm{j}=1}^{\mathrm{N}}\stackrel{\sim }{{\mathrm{f}}_{\mathrm{j}}}}.$$

Following Boruta selection Eq. (1) is adapted and using the indicators and weights demonstrated in Table 6. The scoring of the indicators derives from Table 2.

### The PVI index and classification

The spatial distribution of the PVI can support decision-making regarding funding allocation, reinforcement priorities, choice of type and location of structural measures, priority setting, etc. The aim of the assessment guides the choice of the classification method (e.g. natural breaks, equal intervals, equal counts, standard deviation). Different classification methods lead to different vulnerability maps that consequently will lead to different decisions and strategies for disaster risk reduction.

### Presentation and dissemination

Although PVI and its associated indicators may be demonstrated in tables, bar or line charts, or trend diagrams^16^, in the interest of better communication and collaboration of stakeholders (e.g. local authorities, emergency services, and the public), geographical information systems are considered the ideal tool for storing, analysing, visualising and updating physical vulnerability data.

## Data Availability

The dataset generated and analysed in the present study may become available upon request.
